# The Transforming Growth Factor β Genes and Susceptibility to Musculoskeletal Injuries in a Physically Active Caucasian Cohort

**DOI:** 10.3390/jcm15010358

**Published:** 2026-01-03

**Authors:** Agata Rzeszutko-Bełzowska, Agata Leońska-Duniec

**Affiliations:** 1 Faculty of Physical Culture Sciences, College of Medical Sciences, University of Rzeszow, 35-326 Rzeszow, Poland; 2 Faculty of Physical Education, Gdansk University of Physical Education and Sport, 80-336 Gdansk, Poland; agata.leonska-duniec@awf.gda.pl

**Keywords:** *TGFBI*, *TGFBR3*, *MSTN*, sport, ACL rupture, muscle injury

## Abstract

**Background/Objectives**: Changes in the physiological activity of transforming growth factor-beta (TGF-β) family caused by genetic variability may significantly affect the phenotype of the musculoskeletal system and, consequently, the risk of sports injuries. This study aimed to investigate whether the *TGFBI* (rs1442), *TGFBR3* (rs1805113 and rs1805117), and *MSTN* (rs11333758) polymorphisms, either individually or in combination, were associated with susceptibility to muscle injury, anterior cruciate ligament (ACL) rupture, and other injuries. **Methods**: The study group included 202 physically active Caucasians with reported sport injuries and 133 healthy controls. All the samples were genotyped using real-time polymerase chain reaction (real-time PCR). **Results**: The results revealed that (1) the *TGFBR3* rs1805117 TC genotype was nominally associated with increased ACL injury risk; (2) the *MSTN* rs11333758 heterozygotes was more frequent in the one injury group (vs controls) and in the ACL group, whereas in the multiple vs. one comparison the over-dominant model suggested lower odds for heterozygotes; and (3) the *TGFBI* rs1442 CG genotype was nominally associated with lower odds of fractures, dislocations or sprains. In addition, simultaneous analysis of chosen SNPs revealed interactions between *TGFBR3* rs1805117 and rs1805113, with a nominal association of the rs1805113 G allele with increased injury risk, as did rs11333758 and rs1805113, with a potential effect of rs11333758 on injury status. However, haplotype analysis of the *TGFBR3* SNPs revealed no significant associations. After Bonferroni correction, none of the associations remained statistically significant. **Conclusions**: The results suggested that carrying specific *TGFBI*, *TGFBR3*, and *MSTN* genotypes may be potentially associated with susceptibility to musculoskeletal injuries in a physically active Caucasians.

## 1. Introduction

The adaptive capacity of skeletal muscle and tendons to changing external factors, such as the volume and intensity of exercise, as well as previous injuries and other physiological conditions, is a key element in determining an individual’s overall mobility, athletic ability, and health. The close anatomical connection between musculoskeletal structures and the shared microcirculation and interstitial fluid suggests efficient communication between these tissues mediated by activated transforming growth factor-beta (TGF-β) family members [1]. This family consists of more than 50 molecules that are structurally related, namely, numerous peptide growth and differentiation factors, including activins, bone morphogenetic proteins (BMPs), growth and differentiation factors (GDFs), and three isoforms, namely, TGF-β 1–3. Among them, GDF8, also known as myostatin (MSTN), is a negative regulator of skeletal muscle growth and development [2,3].

Biological processes under the regulation of TGF-β, specifically myoblast and fibroblast cell survival, growth, differentiation, proliferation, metabolism, and death; efficient recycling of damaged contractile proteins; regulation of collagen organization; and muscle extracellular matrix (ECM) remodeling, all induce advantageous changes in the musculotendinous system, which allow for enhanced force transmission throughout the kinetic chain and tissue repair [1,4,5]. However, under certain conditions, such as ischemia‒reperfusion injury, the activation of TGF-β can induce greater ECM strengthening and progressive fibrosis. Thus, TGF-β plays a role in the pathogenesis of fibrosis in numerous organs, as well as in skeletal muscles, where it impairs myocyte function [6,7].

Previous studies have indicated that the proteoglycan betaglycan, TGF-β receptor III (TGFβR3), is able to bind to TGF-β ligands and is implicated in the capture and retention of TGF-β for presentation to signaling receptors [8]. Additionally, the adhesion protein TGF-β induced (TGFβI) plays a key role in collagen deposition [9], whereas MSTN is crucial for the regulation of skeletal muscle growth and differentiation [2]. These studies highlight the importance of these diverse regulatory molecules in the development and healing of muscle and connective tissues and fibrosis. Therefore, changes in the physiological activity of molecules belonging to the TGF-β family caused by genetic variability in the genes encoding them may significantly affect the phenotype of muscles and tendons and, consequently, the risk of sports injuries. To date, few studies have examined single nucleotide polymorphisms (SNPs) within genes encoding TGF-β family members in the context of musculoskeletal soft tissue injuries such as Achilles tendon pathology and anterior cruciate ligament (ACL) ruptures [10,11,12]. Reported findings have been inconsistent, and the relevance of these variants to muscle injury risk remains largely unclear. These inconsistencies likely reflect substantial heterogeneity across studies, including differences in (i) the injury phenotype and case definition, (ii) the specific loci interrogated within the broader TGF-β family pathway, (iii) sample size and statistical power, and (iv) population background and sex composition. In addition, most available association studies have focused on single variants and a single injury outcome, despite the likelihood that injury susceptibility reflects combined genetic effects and may differ across injury subtypes. Here, we address this gap by examining candidate variants in *TGFBI*, *TGFBR3*, and *MSTN* in a physically active Caucasian cohort across distinct injury outcomes (ACL injury, muscle injury, fracture, dislocation, and sprain), and by complementing single-SNP analyses with SNP–SNP interaction testing and haplotype analysis. This approach provides a more integrated assessment of TGF-β–related genetic variability in musculoskeletal injury susceptibility.

Therefore, in the present study, four polymorphisms located in three genes, namely, *TGFBI* (rs1442, G > C, p.Leu217=, synonymous exonic), *TGFBR3* (rs1805113, G > A, p.Phe676=, synonymous exonic and rs1805117, T > C, 3′ untranslated region—3′UTR), and *MSTN* (rs11333758, c.373 + 90delA, intronic deletion), were selected for analysis. These genes were selected based on their well-established roles in muscle and connective-tissue remodeling and on previous studies linking TGF-β–pathway genetic variation to musculoskeletal injury phenotypes. This study aimed to investigate whether these SNPs, either individually or in combination, were associated with susceptibility to muscle injury, ACL rupture, and other conditions, such as fracture, dislocation, or sprain, in a physically active Caucasian cohort. We hypothesized that (1) specific genotypes of selected genes would be individually associated with an increased risk of musculoskeletal injury and (2) the interaction between the studied SNPs would form a specific genotype combination that predisposes its carriers to a greater risk of these injuries.

## 2. Materials and Methods

### 2.1. Ethics

The investigation protocols were conducted ethically according to the World Medical Association Declaration of Helsinki and to the Strengthening the Reporting of Genetic Association studies statement (STREGA). The Bioethics Committee at the District Medical Chamber in Gdansk (No. KB-8/22) granted consent to carry out the research. Informed consent was obtained from all subjects involved in the study.

### 2.2. Participants

The study involved 392 unrelated Caucasians recruited between the years 2022 and 2024. The study group comprised 202 physically active individuals (43 females and 159 males) with muscle and ACL injuries. An additional 57 participants (9 females and 48 males) with other types of injuries were included for correlation analyses. All individuals were physically active and met the same inclusion criteria. No individual was included in more than one group. Detailed participant characteristics are provided in Table 1.

The time spent on exercise of a specific intensity was considered to classify participants as physically active. Individuals who engaged in moderate exercise for at least 150 min per week or vigorous exercise for at least 75 min per week were eligible to participate in the study.

The findings indicated that 4.9% of participants exercised daily, 11.9% exercised six times a week, 15.4% exercised five times a week, 24.6% exercised four times a week, 34.5% exercised three times a week, and 9.8% exercised twice weekly. The respondents’ average training units were: >90 min—11.9%, 60–90 minutes—59.8%, 30–60 minutes—28.1%. Most of them were representatives of endurance disciplines, mainly long-distance running. The most common muscle injuries concerned thigh muscles—61.9%, feet—37.3%, calves—25.2%, upper limb girdle—19%, and hips—6.3%. Additionally, 28% of the study group had other injuries, including fractures, dislocations, and sprains. People with confirmed ACL injuries were also qualified for the study (n = 60, 19 females and 43 males).

The control group comprised 133 healthy, unrelated individuals (47 females and 86 males) with no history of soft tissue injuries. Group allocation was based on responses to the International Physical Activity Questionnaire (IPAQ). Similarly to the study group, control participants engaged in at least 150 minutes of moderate-intensity exercise per week or at least 75 minutes of vigorous-intensity exercise per week.

### 2.3. DNA Analyses

Total genomic DNA was extracted from buccal swabs (Copan FLOQSwabs, Interpath, Murrieta, Australia) of each participant qualified for the study using the directions provided with a High Pure PCR Template Preparation Kit (Roche, Basel, Switzerland). All samples were genotyped twice, applying TaqMan^®^ Pre-De-signed SNP Genotyping Assays (Applied Biosystems, Waltham, MA, USA) on a C1000 Touch Thermal Cycler (BioRad, Hercules, CA, USA). To discriminate the *TGFBI* rs1442, *TGFBR3* rs1805113 and rs1805117, as well as *MSTN* rs11333758 alleles, the following assays ID were used: C___2563834_20, C___1931721_10, C___8368244_10, and C___175825166_10, respectively. The genotyping mixture per reaction included 2.5 μL of TaqPath™ ProAmp™ Master Mix (Applied Biosystems, Waltham, MA, USA), 0.25 μL of assay mix (10×), and 1.0 μL of distilled water with 1 μL of genomic DNA. The real-time PCR conditions were as follows: 30 s of pre-read in 60 °C, 5 min of preliminary denaturation in 95 °C, cycling 5 s of denaturation in 95 °C, 30 s of primer hybridization and elongation in 60 °C, repeated in 40 cycles, 30 s of terminal elongation in 60 °C. The obtained products were visualized and analyzed using CFX Maestro 4.0 Software (BioRad, Hercules, CA, USA).

### 2.4. Statistical Analyses

All of the analyses have been conducted in R version 4.4.2 (R Foundation for Statistical Computing, Vienna, Austria). Prior to the analysis of polymorphisms, the control group was compared with the study group with regard to additional factors that could interact with genetic variants (age, sex, body weight, height, body mass index—BMI) referred to as covariates. Numerical covariates were compared using Student’s t-test, while the categorical variable (sex) was assessed with the chi-square test. As sex was significantly differentially distributed between groups, it has been used as a covariate in all association models.

Information on variant positions, ID, consequences, and frequency in the non-Finnish European population has been downloaded from the gnomAD database v. 4.1.0. For clarity, non-reference allele frequencies are reported with regard to the GRCh38 human reference genome; therefore, these are not always minor allele frequencies. 

The association and interaction analysis of polymorphisms and haplotypes with the occurrence of sport-related injuries, as well as the analysis of variant frequencies and compliance with Hardy–Weinberg Equilibrium (HWE), were conducted using the SNPassoc library (version 2.1–0). Each variant was first analyzed in terms of its association with any of the remaining covariates after adjustment for sex with a generalized linear model (GLM). Covariates, apart from sex, were not included in the main association models as they were not significantly associated (nominal *p* < 0.05) with any of the polymorphisms.

Associations of polymorphisms were investigated using generalized linear models, and interaction analyses employed the log-likelihood ratio test (LTR). Pairs of variants showing nominally significant interactions in the LRT were further analyzed with generalized linear models.

Haplotype analysis was performed for variants within *TGFBR3* using the haplo.stats library and consisted of two stages. The first stage involved identifying the most probable haplotypes using the expectation-maximization (EM) algorithm. The second stage involved testing the association of each haplotype with muscle injuries GLM. In each association analysis, 6 comparative analyses (log-Additive, Codominant, Dominant, Overdominant Recessive, and Codominant model with epistasis) were performed, resulting in nominal *p*-values reported.

In each association analysis, both nominal *p*-values and *p*-values adjusted for multiple comparisons using the Bonferroni correction are reported, apart from the interaction results, where 6 comparisons were made.

## 3. Results

The frequencies of the analyzed SNPs, provided for the non-reference allele in each of the studied subgroups, were compared with values from the population database (GnomAD, Table S1).

Each of the variants was also analyzed for accordance HWE (Table S2). None of the SNPs violated HWE according to the Bonferroni-corrected *p*-value threshold (adjusted *p* < 0.05) in the full study group. SNP rs1442 showed significant deviation from HWE (nominal *p* < 0.05) in the group with other injuries (*p* = 0.02), where the genotypes are distributed equally (GG = 19, GC = 19, CC = 19), which is not expected under HWE (expected counts: 14, 29, and 14, respectively).

### 3.1. Individual SNP Analysis

**Figure 1:**
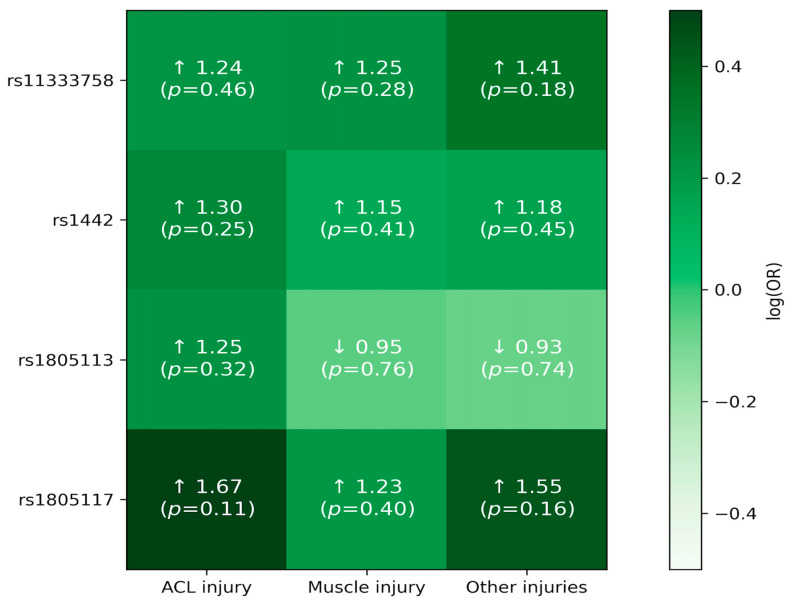
Visual summary of SNP associations across injury subgroups (additive model). Heatmap showing the direction and magnitude of associations for each SNP across injury subgroups, expressed as log (OR) from additive-model logistic regression. Darker green indicates a larger positive log (OR), whereas lighter green indicates a more negative log (OR) (scale centered at 0). Cell annotations report OR and *p*-value; arrows indicate effect direction (↑ OR > 1, ↓ OR < 1).

**Figure 2:**
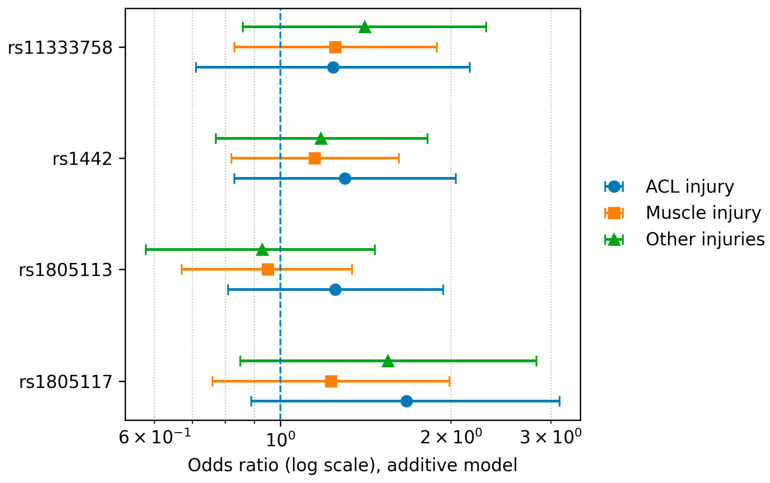
SNP associations across injury subgroups (additive model). Dot plot showing odds ratios (ORs) with 95% confidence intervals (CIs) for each SNP under the additive genetic model across injury subgroups (ACL injury, muscle injury, and other injuries). The dashed vertical line indicates OR = 1; markers denote subgroups.

Each SNP was analyzed for association with muscle injuries (control group vs. any injury). None of the SNPs were significantly differently distributed between the tested groups under any of the models (Table S3). Figure 1 offers a visual overview of the association patterns across SNPs and injury subgroups, and Figure 2 highlights the effect sizes (ORs with 95% CIs) for direct comparison. Full numerical results are provided in Table 2 and Table 3.

This analysis revealed multiple nominally significant associations, which we report below. For rs1805117, the heterozygous genotype (TC) was associated with higher odds of ACL injury compared with the reference genotype (TT) under the codominant model (OR = 2.05, 95% CI = 1.05–3.99; *p* = 0.047). Consistently, the overdominant model (TC vs. TT + CC) also indicated increased odds of ACL injury (OR = 2.10, 95% CI = 1.08–4.08; *p* = 0.029).

For *MSTN* rs11333758, we see an association when controls are compared with participants with a single injury under the codominant (A– vs. AA: OR = 1.91, 95% CI = 1.06–3.45; *p* = 0.031) and overdominant (A– vs. AA/– –: OR = 2.01, 95% CI = 1.12–3.60; *p* = 0.019) models. When comparing participants with multiple injuries to those with one injury, an overall genotype effect was observed under the codominant (*p* = 0.017), recessive (*p* = 0.024), and overdominant (*p* = 0.034) models. In this comparison, heterozygotes showed lower odds of multiple injuries (overdominant: A– vs. AA/– –: OR = 0.53, 95% CI = 0.29–0.96), whereas the rare homozygous genotype (– –) showed higher odds (codominant:—– vs. AA: OR = 5.49, 95% CI = 0.67–44.98; recessive: –– vs. AA/A–: OR = 6.80, 95% CI = 0.84–54.91), noting the wide CIs due to sparse counts. The same *MSTN* SNP showed an association with ACL injury under the codominant and overdominant models (codominant: A– vs. AA: OR = 1.88, 95% CI = 0.98–3.62; *p* = 0.017; overdominant: A– vs. AA/––: OR = 2.02, 95% CI = 1.05–3.87; *p* = 0.035). The recessive model also yielded a nominal signal (*p* = 0.032), but the rare homozygous genotype (– –) was absent among ACL cases, precluding a stable OR estimate.

For *TGFBI* rs1442, heterozygosity (CG) was nominally associated with lower odds of other injuries (fractures, dislocations, or sprains) compared with homozygotes (CC + GG) under the overdominant model (OR = 0.49, 95% CI = 0.25–0.95; *p* = 0.031), suggesting relatively higher odds among CC and GG carriers.

After applying the Bonferroni correction for multiple comparisons, none of the previously reported nominal associations remained statistically significant.

Groups with over 10 participants were included. As the same individuals could have had multiple injuries, only comparisons with the control group were possible. At the head of each table section the SNP and the comparison are listed. *p*-values were adjusted using the Bonferroni method and assuming 4 independent comparisons. OR—odds ratio, CI—confidence interval

### 3.2. Epistasis Analysis

**Figure 3:**
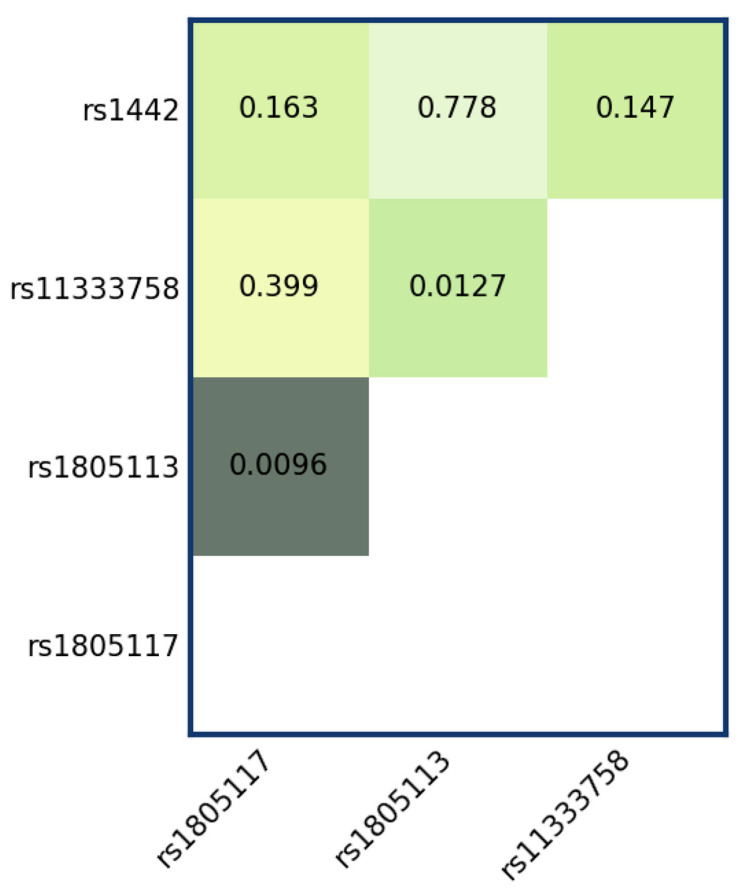
Results of tests for interactions between tested SNPs in the codominant model. Nominal *p*-values for each comparison are reported on the heatmap. The saturation of each rectangle with green color is proportional to the −log10 (*p*-value).

We moreover investigated SNP-SNP pairwise interactions using the LTR for each comparison, assuming a codominant model (Figure 3). Under this model, each SNP’s genotype is treated as a categorical factor (no allele grouping), so the interaction encompasses all genotype combinations. This analysis provided nominal evidence of SNP–SNP interactions between rs1805117 and rs1805113 (*p* = 0.0096; adjusted *p* = 0.057) and between rs11333758 and rs1805113 (*p* = 0.0127; adjusted *p* = 0.076). However, these signals did not remain statistically significant after multiple-testing correction. Overall, the results suggest that the joint effects of these SNP pairs may deviate from what would be expected under an independence (no-interaction) assumption, warranting replication in an independent cohort. Therefore, we analysed the pairwise interaction between these SNPs further using linear models (Table 4). 

For the *TGFBR3* rs1805117 and rs1805113 SNPs, after adjusting for rs1805117 genotype (and sex), rs1805113 was nominally associated with injury status (*p* = 0.009). Compared with AA, carriers of rs1805113 AG and GG showed OR = 1.31 (95% CI: 0.73–2.37) and OR = 1.10 (95% CI: 0.49–2.45), respectively (noting sparse cells for some genotype combinations). After adjustment for rs1805113 genotype, rs1805117 was not associated with injury status (*p* = 0.134). A haplotype analysis of rs1805117–rs1805113 did not reveal significant associations (T–G: OR = 1.13, 95% CI: 0.79–1.61, *p* = 0.495; C–A: OR = 1.15, 95% CI: 0.71–1.87, *p* = 0.564; Table 5).,For the rs11333758–rs1805113 pair, rs11333758 remained associated with injury status after adjustment for rs1805113 (*p* = 0.0039); the strongest effect was observed among rs1805113 GG carriers, where rs11333758 heterozygotes (A–) had OR = 6.94 (95% CI: 1.73–27.95) compared with rs11333758 AA. Conversely, rs1805113 remained associated after adjustment for rs11333758 (*p* = 0.002); among rs11333758 A—carriers, rs1805113 GG showed OR = 4.41 (95% CI: 1.07–18.25) compared with rs1805113 AA.

## 4. Discussion

Proper TGF-β signaling is important for maintaining health, whereas dysfunctional signaling can lead to several pathological conditions, including disorders of developmental defects, aberrant healing, inflammation, fibrosis, infectious diseases, muscular dystrophy, and cancer [4,13]. Therefore, SNPs located in genes encoding the TGF-β family members and their receptors may affect protein expression and/or function and, consequently, TGF-β signaling. This heterogeneity may be an advantageous or disadvantageous factor for various biological processes, including physiological performance, and may be associated with different posttraining changes and an individual’s susceptibility to skeletal muscle and tendon injuries.

This is the first study to attempt to identify potential genetic associations of *TGFBI* (rs1442), *TGFBR3* (rs1805113 and rs1805117), and *MSTN* (rs11333758), either individually or in combination, with different musculoskeletal injuries, such as ACL rupture, muscle injuries, as well as fractures, dislocations, and sprains. The main finding was that specific *TGFBI*, *TGFBR3*, and *MSTN* genotypes may be associated with susceptibility to musculoskeletal injuries in a physically active Caucasian population. The results provided support for the first hypothesis and showed that i) the *TGFBR3* rs1805117 TC genotype was nominally associated with a greater risk of ACL injury; ii) the *MSTN* rs11333758 heterozygotes was more frequent in the one injury group (vs controls) and in the ACL group, whereas in the multiple vs. one comparison the overdominant model suggested lower odds for heterozygotes; and iii) the *TGFBI* rs1442 CG genotype was nominally associated with lower odds of fractures, dislocations or sprains. In addition, simultaneous analysis of chosen SNPs suggested interactions between *TGFBR3* rs1805117 and rs1805113, with a nominal association between the rs1805113 G allele and increased injury risk. Interactions between rs11333758 and rs1805113 were also shown, with a nominal effect of rs11333758 on injury status, thus providing support for our second hypothesis. However, subsequent haplotype analysis of the *TGFBR3* SNPs revealed no significant associations of any of the haplotypes with injury status. Because multiple SNPs, genetic models, and injury phenotypes were tested, we applied a Bonferroni correction; associations were nominal and did not remain significant after correction, and should therefore be regarded as exploratory. Moreover, the observed effects may reflect linkage disequilibrium with other functional variants and cannot be interpreted as causal. Replication in larger, independent cohorts and functional studies will be needed to confirm these signals and clarify underlying mechanisms.

In a previous study including 459 European Caucasian ancestry participants from South Africa (249 with surgically diagnosed ACL ruptures and 210 asymptomatic control participants), Laguette and coworkers (2020) revealed novel implications of TGFBR3 and TGFBI in modulating the risk of ACL injury. They described significant associations of the *TGFBR3* rs1805113 G allele with a decreased risk of ACL rupture in all participants and females, as well as an association between the *TGFBI* rs1442 CC genotype and a greater risk of this injury in the female cohort. In addition, haplotype analysis supported these findings and indicated that the A;T (rs1805113, 1805117, respectively) haplotype was associated with an increased risk of ACL injury. Conversely, the G;C haplotype was linked to a decreased risk [10]. Posthumus and coworkers (2010) selected the *TGFB1* rs1800469 and *GDF5* rs143383 polymorphisms for analysis and investigated their relationship with Achilles tendon pathology in a group of 172 participants with this injury and 235 asymptomatic control individuals from Australia and South Africa. This study suggested that the *GDF5* rs143383 TT genotype was associated with twice the risk of developing Achilles tendon pathology. However, there were no significant differences in *TGFB1* rs1800469 genotype or allele frequency between the study and control groups [11]. Chen and coauthors (2015) confirmed the usefulness of the *GDF5* rs143383 polymorphism for assessing the risk of ACL rupture in the Chinese population. They revealed that the frequency of the TT genotype and T allele tended to be greater in the ACL rupture group than in the control group. In addition, participants carrying the TT genotype expressed lower levels of *GDF5* mRNA than C carriers among those with ACL rupture. Conversely, in a study conducted on 126 Caucasians with ACL rupture and 214 controls, Raleigh et al. (2013) reported no significant differences in the frequency of the *GDF5* rs143383 genotype (*p* = 0.396) or allele (*p* = 0.810) between the two groups [12].

Although the *MSTN* rs11333758 SNP has not been studied in the context of musculoskeletal injuries, its relationship with sports predispositions has been described [13,14]. Animal studies have shown that loss or decrease in myostatin activity reduces muscle quality, defined as the capacity to produce force at a given muscle mass or volume, which may alter the risk of soft tissue injuries. Potential mechanisms involve the suppression of mitochondrial biogenesis and/or muscle protein turnover as a result of myostatin inhibition [15]. This study was the first to demonstrate a potential association of rs11333758 with a modulated risk of single and multiple injuries, as well as susceptibility to ACL injury, underscoring the need to continue research in this direction.

Possible biological mechanisms underlying the association between the TGF-β signaling pathway and susceptibility to musculoskeletal soft-tissue injuries have been described. TGF-β is a bifunctional regulator that functions in a context- and cell type-dependent manner. Cell survival, proliferation, differentiation, growth, adhesion, migration, metabolism, and apoptosis are all under this regulation [3,4]. In addition, proteins belonging to this family modulate fibroblast behavior and the process of myofibroblast transformation. The TGF-β pathway supports the maintenance of the ECM by increasing the production of matrix proteins and stimulating the expression of ECM-related and fibrogenic genes, including those encoding type I collagen and connective tissue growth factor (CTGF). Simultaneously, it inhibits enzymes responsible for ECM breakdown, such as matrix metalloproteinases (MMPs) [16,17]. Activation of this pathway promotes the accumulation of collagen, a key element in wound healing. However, in response to pathological stimuli such as ischemia‒reperfusion injury, this process can become enhanced, leading to excessive ECM accumulation and ongoing fibrosis [18]. Therefore, TGF-β, via the activation of both canonical (Smad-based) and noncanonical (non-Smad-based) signaling pathways, is implicated in the pathogenesis of fibrosis in numerous organs and tissues, including skeletal muscles. It manifests through excessive ECM production and inhibition of ECM degradation in muscle cross-sections, with increased collagen leading to decreased muscle fiber size, greater stiffness, and a reduction in maximum isometric force production [6]. This fibrosis reduces the volume of functional muscle tissue available for therapeutic intervention and regeneration [19]. Notably, TGF-β can induce skeletal muscle fibrosis and reduce force-generating capacity independent of injuries or disease [20,21]. Published data may indicate a significant contribution of genetic factors influencing the TGF-β signaling pathway and explain a potential link between the genes encoding members of this family and the risk of musculoskeletal soft-tissue injuries.

On the basis of the literature data and our previous research, four polymorphisms located in three genes were selected for this analysis. Unfortunately, little is known about the functional significance of these polymorphisms. The *TGFBR3* rs1805117 SNP, situated within the 3′UTR, is close to sites essential for the formation of disulfide bonds and glycosylation. This region of the gene may play a key role in regulating receptor expression and function [22]. Interestingly, two other SNPs are synonymous variants that do not affect the amino acid change in the protein sequence, but their effects on expression at various levels, including RNA stability and translation, have been suggested. The first *TGFBR3* rs1805113 (Phe676Phe) variant results in differential codon usage for Phe_TTT and Phe_TTC. According to previous results, Phe_TTC is highly expressed, indicating that the infrequency of the C allele could lead to lower expression. In addition, a different frequency between Phe_TTT and Phe_TTC depending on gene localization has been observed, suggesting codon-mediated translational control between Phe_TTT and Phe_TTC [23]. The second *TGFBI* rs1442 (Leu217Leu) polymorphism is adjacent to sites significant for calcium binding and within a fasciclin-like domain, which is a cell adhesion domain common to many secreted and membrane-anchored proteins [24]. The last *MSTN* (rs11333758, c.373 + 90delA) polymorphism is the deletion of one of three adenines (AAA→AA) at positions 88–90 bp in the first intron. The occurrence of the alternative codons Leu_GAG and Leu_GAC, as well as the deletion or insertion of adenine in the noncoding sequence, may also have an impact on expression, although this has not been clearly described.

Several limitations should be acknowledged. First, injury status was largely based on self-report; although we captured injury type, we did not systematically collect or verify injury severity, timing, clinical confirmation (except where available), rehabilitation details, or time to return to sport. These factors can influence both continued physical activity and the likelihood of recurrent injuries, and may therefore introduce misclassification. Second, physical activity and group allocation were based on IPAQ and eligibility thresholds. While we recorded training frequency and typical session duration, we did not quantify objective training load (e.g., intensity distribution, pace/power/heart rate, competition exposure, surface, footwear, workload), which may vary substantially even within an endurance-dominant cohort and may confound genetic associations. Third, although most participants practiced endurance sports, predominantly long-distance running, residual heterogeneity in training practices and sport-specific risk factors could not be fully controlled. Fourth, controls were defined as physically active individuals without a self-reported history of soft-tissue injuries using the same IPAQ-based approach. However, we could not exclude unmeasured differences between cases and controls in factors such as prior minor injuries, biomechanics, strength training, recovery practices, or medical history. Finally, multiple SNPs, genetic models, and injury phenotypes were tested; thus, some nominal associations may represent false positives despite correction procedures, and the findings should be considered exploratory pending replication in larger, well-phenotyped cohorts with standardized injury definitions and objective exposure monitoring.

## 5. Conclusions

The main finding of the present study was that the specific *TGFBI* rs1442, *TGFBR3* rs1805113, rs1805117, and *MSTN* rs11333758 genotypes may be associated with susceptibility to musculoskeletal injuries in a physically active Caucasian population. Although these polymorphic sites are interesting genetic markers of sports injuries, future studies with larger sample sizes and different populations will be necessary to confirm these results and reveal the genetic background of this type of injury. Future practical applications of this type of study will include developing individual training programs and other protective elements on the basis of athletes’ genetic predispositions, preserving athlete health with a high genetic risk of musculoskeletal injuries.

## Figures and Tables

**Table 1:** Characteristics of the study participants.

Group	Number of Participants	Number of Men	Number of Women	Average Age	Average Height	Average Body Weight	BMI
Main groups							
Control group	133	86	47	36.90	173.53	71.65	23.37
Study group	202	159	43	36.54	175.36	74.00	25.07
Division according to the number of injuries							
Single	26	19	7	36.61	173.61	72.51	32.74
Multiple	116	97	19	35.94	176.10	74.59	23.90
Division by type of injury							
Muscle injury	142	116	26	36.07	175.64	74.21	25.52
ACL injury	60	43	17	37.66	174.70	73.50	24.00
Other injury	57	48	9	35.71	175.54	74.75	24.08

**Table 2:** Detailed results of association analysis of selected SNPs with different numbers of muscle injuries.

SNP	rs1805117				SNP	rs1805117			
Comparison	No Injuries vs. One Injury				Comparison	No Injuries vs. More Than One Injury			
Model	*p*-Value (*p*-Value Adjusted)	Genotype	OR	95% CI	Model	*p*-Value (*p*-Value Adjusted)	Genotype	OR	95% CI
codominant	0.41 (1)	TT	1		codominant	0.68 (1)	TT	1.0	
		TC	1.50	0.81–2.78			TC	1.24	0.7–2.22
		CC	0.76	0.07–8.67			CC	1.67	0.26–10.5
dominant	0.22 (0.89)	TT	1		dominant	0.41 (1)	TT	1.0	
		TC-CC	1.46	0.80–2.67			TC-CC	1.27	0.72–2.23
recessive	0.74 (1)	TT-TC	1		recessive	0.63 (1)	TT-TC	1.0	
		CC	0.67	0.06–7.56			CC	1.57	0.25–9.8
overdominant	0.19 (0.74)	TT-GG	1.0		overdominant	0.41 (1)	TT-CC	1.0	
		TC	1.51	0.82–2.79			TC	1.22	0.69–2.18
additive	0.29 (1)	0,1,2	1.35	0.77–2.37	additive	0.38 (1)	0,1,2	1.26	0.76–2.09
SNP	rs1805117				SNP	rs1805113			
comparison	one injury vs. more injuries				comparison	no injuries vs. one injury			
model	*p*-value (*p*-value adjusted)	genotype	OR	95% CI	model	*p*-value (*p*-value adjusted)	genotype	OR	95% CI
codominant	0.61 (1)	TT	1.0		codominant	0.66 (1)	AA	1	
		TC	0.8	0.44–1.48			AG	0.94	0.49–1.8
		CC	2.03	0.2–20.22			GG	1.31	0.61–2.83
dominant	0.58 (1)	TT	1.0		dominant	0.86 (1)	AA	1	
		TC-CC	0.85	0.46–1.54			AG-GG	1.05	0.58–1.91
recessive	0.48 (1)	TT-TC	1.0		recessive	0.38 (1)	AA-AG	1	
		CC	2.18	0.22–21.51			GG	1.36	0.69–2.68
overdominant	0.44 (1)	TT-CC	1.0		overdominant	0.57 (1)	AA-GG	1	
		TC	0.79	0.43–1.45			AG	0.85	0.48–1.5
additive	0.76 (1)	0,1,2	0.92	0.53–1.58	additive	0.54 (1)	0,1,2	1.13	0.77–1.65
SNP	rs1805113				SNP	rs1805113			
comparison	no injuries vs. more than one injury				comparison	one injury vs. more injuries			
model	*p*-value (*p*-value adjusted)	genotype	OR	95% CI	model	*p*-value (*p*-value adjusted)	genotype	OR	95% CI
codominant	0.58 (1)	AA	1		codominant	0.19 (0.77)	AA	1	
		AG	1.23	0.69–2.19			AG	1.3	0.68–2.51
		GG	0.86	0.4–1.85			GG	0.64	0.29–1.45
dominant	0.68 (1)	AA	1		dominant	0.86 (1)	AA	1	
		AG-GG	1.12	0.65–1.93			AG-GG	1.06	0.57–1.94
recessive	0.44 (1)	AA-AG	1		recessive	0.10 (0.41)	AA-AG	1	
		GG	0.76	0.39–1.51			GG	0.55	0.27–1.13
overdominant	0.33 (1)	AA-GG	1		overdominant	0.14 (0.57)	AA-GG	1	
		AG	1.29	0.77–2.16			AG	1.54	0.86–2.74
additive	0.88 (1)	0,1,2	0.97	0.67–1.41	additive	0.42 (1)	0,1,2	0.85	0.57–1.27
SNP	rs11333758				SNP	rs11333758			
comparison	no injuries vs. one injury				comparison	no injuries vs. more than one injury			
model	*p*-value (*p*-value adjusted)	genotype	OR	95% CI	model	*p*-value (*p*-value adjusted)	genotype	OR	95% CI
codominant	0.031 (0.12)	AA	1		codominant	0.56 (1)	AA	1	
		A–	1.91	1.06–3.45			A–	1.09	0.62–1.92
		– –	0.31	0.04–2.68			– –	1.75	0.58–5.25
dominant	0.074 (0.3)	AA	1		dominant	0.55 (1)	AA	1	
		A– –	1.69	0.95–2.99			A––– –	1.18	0.69–2.01
recessive	0.13 (0.53)	AA–A–	1		recessive	0.33 (1)	AA–A–	1	
		– –	0.24	0.03–2.06			– –	1.7	0.58–5.03
overdominant	0.019 (0.077)	AA– –	1		overdominant	0.91 (1)	AA– –	1	
		A–	2.01	1.12–3.6			A–	1.03	0.59–1.81
additive	0.270 (1)	0,1,2	1.32	0.8–2.18	additive	0.39 (1)	0,1,2	1.2	0.79–1.84
SNP	rs11333758				SNP	rs1442			
comparison	one injury vs. more than one injury				comparison	no injuries vs. one injury			
model	*p*-value (*p*-value adjusted)	genotype	OR	95% CI	model	*p*-value (*p*-value adjusted)	genotype	OR	95% CI
codominant	0.017 (0.067)	AA	1		codominant	0.26 (1)	CC	1	
		A–	0.58	0.32–1.06			CG	1.13	0.57–2.26
		– –	5.49	0.67–44.98			GG	1.85	0.83–4.11
dominant	0.25 (1)	AA	1		dominant	0.38 (1)	CC	1	
		A– –	0.71	0.4–1.27			CG-GG	1.33	0.69–2.56
recessive	0.024 (0.098)	AA–A–	1		recessive	0.11 (0.44)	CC-CG	1	
		– –	6.8	0.84–54.91			GG	1.7	0.89–3.27
overdominant	0.034 (0.14)	AA– –	1		overdominant	0.54 (1)	CC-GG	1	
		A–	0.53	0.29–0.96			CG	0.84	0.48–1.47
additive	0.84 (1)	0,1,2	0.95	0.59–1.54	additive	0.13 (0.52)	0,1,2	1.36	0.91–2.03
SNP	rs1442				SNP	rs1442			
comparison	no injuries vs. more than one injury				comparison	one injury vs. more than one injury			
model	*p*-value (*p*-value adjusted)	genotype	OR	95% CI	model	*p*-value (*p*-value adjusted)	genotype	OR	95% CI
codominant	0.58 (1)	CC	1		codominant	0.60 (1)	CC	1	
		CG	0.84	0.46–1.53			CG	0.77	0.38–1.56
		GG	1.18	0.57–2.42			GG	0.67	0.31–1.47
dominant	0.81 (1)	CC	1		dominant	0.35 (1)	CC	1	
		CG–GG	0.93	0.53–1.65			CG-GG	0.73	0.38–1.41
recessive	0.39 (1)	CC-CG	1		recessive	0.48 (1)	CC-CG	1	
		GG	1.31	0.71–2.43			GG	0.79	0.42–1.5
overdominant	0.35 (1)	CC–GG	1		overdominant	0.84 (1)	CC-GG	1	
		CG	0.78	0.47–1.31			CG	0.94	0.53–1.68
additive	0.72 (1)	0,1,2	1.07	0.75–1.53	additive	0.32 (1)	0,1,2	0.82	0.56–1.21

At the head of each table section the SNP and the comparison are listed, *p*-values, and OR–odds ratio.

**Table 3:** Detailed results of the association analysis of selected SNPs with different types of muscle injuries.

SNP	rs1805117				SNP	rs1805117			
Comparison	Controls vs. Muscle Strain or Tear				Comparison	Control vs. Other Injuries (Sprains, Breaks, and Twists)			
Model	*p*-Value (*p*-Value Adjusted)	Genotype	OR	95% CI	Model	*p*-Value (*p*-Value Adjusted)	Genotype	OR	95% CI
codominant	0.68 (1)	TT	1		codominant	0.37 (1)	TT	1	
		TC	1.18	0.68–2.07			TC	1.54	0.76–3.08
		CC	1.82	0.32–10.63			CC	2.5	0.33–18.98
dominant	0.47 (1)	TT	1		dominant	0.18 (0.72)	TT	1	
		TC–CC	1.22	0.71–2.1			TC–CC	1.59	0.81–3.14
recessive	0.53 (1)	TT–TC	1		recessive	0.45 (1)	TT–TC	1	
		CC	1.74	0.31–9.81			CC	2.19	0.29–16.47
overdominant	0.59 (1)	TT–CC	1		overdominant	0.26 (1)	TT–CC	1	
		TC	1.16	0.67–2.03			TC	1.49	0.74–2.97
additive	0.40 (1)	0,1,2	1.23	0.76–1.99	additive	0.16 (0.63)	0,1,2	1.55	0.85–2.83
SNP	rs1805117				SNP	rs1805113			
comparison	controls vs. ACL injury				comparison	controls vs. muscle strain or tear			
model	*p*-value (*p*-value adjusted)	genotype	OR	95% CI	model	*p*-value (*p*-value adjusted)	genotype	OR	95% CI
codominant	0.047 (0.19)	TT	1		codominant	0.81 (1)	AA	1	
		TC	2.05	1.05–3.99			AG	1.07	0.62–1.86
		CC	0	NA			GG	0.85	0.42–1.75
dominant	0.053 (0.21)	TT	1		dominant	0.98 (1)	AA	1	
		TC–CC	1.92	0.99–3.72			AG–GG	1.01	0.6–1.69
recessive	0.19 (0.77)	TT–TC	1		recessive	0.54 (1)	AA–AG	1	
		CC	0	NA			GG	0.82	0.43–1.56
overdominant	0.029 (0.12)	TT–CC	1		overdominant	0.62 (1)	AA–GG	1	
		TC	2.1	1.08–4.08			AG	1.13	0.69–1.85
additive	0.11 (0.43)	0,1,2	1.67	0.89–3.11	additive	0.76 (1)	0,1,2	0.95	0.67–1.34
SNP	rs1805113				SNP	rs1805113			
comparison	control vs. other injuries (sprains, breaks, and twists)				comparison	controls vs. ACL injury			
model	*p*-value (*p*-value adjusted)	genotype	OR	95% CI	model	*p*-value (*p*-value adjusted)	genotype	OR	95% CI
codominant	0.15 (0.6)	AA	1		codominant	0.51 (1)	AA	1	
		AG	1.54	0.76–3.15			AG	1.49	0.71–3.11
		GG	0.64	0.22–1.85			GG	1.53	0.62–3.75
dominant	0.49 (1)	AA	1		dominant	0.25 (1)	AA	1	
		AG–GG	1.27	0.64–2.52			AG–GG	1.5	0.75–3.01
recessive	0.12 (0.48)	AA–AG	1		recessive	0.69 (1)	AA–AG	1	
		GG	0.49	0.19–1.28			GG	1.2	0.56–2.58
overdominant	0.078 (0.31)	AA–GG	1		overdominant	0.49 (1)	AA–GG	1	
		AG	1.78	0.93–3.38			AG	1.25	0.67–2.35
additive	0.74 (1)	0,1,2	0.93	0.58–1.47	additive	0.32 (1)	0,1,2	1.25	0.81–1.94
SNP	rs11333758				SNP	rs11333758			
comparison	controls vs. muscle strain or tear				comparison	control vs. other injuries (sprains, breaks and twists)			
model	*p*-value (*p*-value adjusted)	genotype	OR	95% CI	model	*p*-value (*p*-value adjusted)	genotype	OR	95% CI
codominant	0.84 (1)	AA	1		codominant	0.24 (0.96)	AA	1	
		A–	1.21	0.71–2.08			A–	1.09	0.53–2.21
		– –	1.67	0.57–4.86			– –	2.76	0.85–8.99
dominant	0.35 (1)	AA	1		dominant	0.40 (1)	AA	1	
		A– –	1.28	0.77–2.13			A– –	1.32	0.69–2.53
recessive	0.40 (1)	AA–A–	1		recessive	0.094 (0.38)	A–A–	1	
		– –	1.56	0.54–4.49			– –	2.69	0.85–8.54
overdominant	0.58 (1)	AA– –	1		overdominant	0.92 (1)	AA– –	1	
		A–	1.16	0.68–1.97			A–	0.96	0.48–1.93
additive	0.28 (1)	0,1,2	1.25	0.83–1.89	additive	0.18 (0.71)	0,1,2	1.41	0.86–2.31
SNP	rs11333758				SNP	rs1442			
comparison	controls vs. ACL injury				comparison	controls vs. muscle strain or tear			
model	*p*-value (*p*-value adjusted)	genotype	OR	95% CI	model	*p*-value (*p*-value adjusted)	genotype	OR	95% CI
codominant	0.017 (0.067)	AA	1		codominant	0.36 (1)	CC	1	
		A–	1.88	0.98–3.62			CG	0.87	0.48–1.56
		– –	0	NA			GG	1.37	0.69–2.72
dominant	0.14 (0.57)	AA	1		dominant	0.96 (1)	CC	1	
		A– –	1.62	0.85–3.07			CG–GG	1.01	0.59–1.75
recessive	0.032 (0.18)	AA–A–	1		recessive	0.18 (0.72)	CC–CG	1	
		– –	0	NA			GG	1.49	0.83–2.67
overdominant	0.035 (0.14)	AA– –	1		overdominant	0.27 (1)	CC–GG	1	
		A–	2.02	1.05–3.87			CG	0.76	0.46–1.24
additive	0.46 (1)	0,1,2	1.24	0.71–2.16	additive	0.41 (1)	0,1,2	1.15	0.82–1.62
SNP	rs1442				SNP	rs1442			
comparison	control vs. other injuries (sprains, breaks, and twists)				comparison	controls vs. ACL injury			
model	*p*-value (*p*-value adjusted)	genotype	OR	95% CI	model	*p*-value (*p*-value adjusted)	genotype	OR	95%CI
codominant	0.067 (0.27)	CC	1		codominant	0.31 (1)	CC	1	
		CG	0.58	0.27–1.24			CG	0.94	0.44–2.02
		GG	1.45	0.63–3.31			GG	1.7	0.71–4.07
dominant	0.58 (1)	CC	1		dominant	0.71 (1)	CC	1	
		CG–GG	0.83	0.42–1.64			CG–GG	1.14	0.56–2.34
recessive	0.063 (0.25)	CC–CG	1		recessive	0.13 (0.50)	CC–CG	1	
		GG	1.98	0.97–4.04			GG	1.77	0.86–3.65
overdominant	0.031 (0.12)	CC–GG	1		overdominant	0.33 (1)	CC–GG	1	
		CG	0.49	0.25–0.95			CG	0.73	0.39–1.38
additive	0.45 (1)	0,1,2	1.18	0.77–1.82	additive	0.25 (1)	0,1,2	1.3	0.83–2.04

**Table 4:** Detailed interaction results for pairs of SNPs with a nominally significant interaction detected in the baseline analysis.

rs1805113 × rs1805117 interaction results (*p* = 0.0096239) Association of rs1805113 with rs1805117 (*p* = 0.0096)						
rs1805117	rs1805113	ctrl count	injured count	OR	lower 95% CI	upper 95% CI
TT	AA	44	55	1.00	-	-
CT	AA	33	55	1.31	0.73	2.37
CC	AA	14	19	1.10	0.49	2.45
TT	AG	0	9	-	-	-
CT	AG	24	38	1.26	0.65	2.42
CC	AG	8	15	1.39	0.54	3.62
TT	GG	0	0	-	-	-
CT	GG	0	0	-	-	-
CC	GG	2	4	1.48	0.25	8.57
Association of rs1805117 with injury after adjusting for rs1805113 genotype (*p* = 0.13397)						
rs1805117	rs1805113	ctrl count	injured count	OR	lower 95% CI	upper 95% CI
TT	AA	44	55	1.00		
TT	AG	0	9	-	-	-
TT	GG	0	0	-	-	-
CT	AA	33	55	1.00	-	-
C/T	AG	24	38	0.96	0.49	1.88
CT	GG	0	0	-	-	-
CC	AA	14	19	1.00	-	-
CC	AG	8	15	1.27	0.42	3.87
CC	GG	2	4	1.35	0.21	8.57
Association of rs1805113 with injury after adjusting for rs1805117 genotype (*p* = 0.0093438)						
rs1805117	rs1805113	ctrl count	injured count	OR	lower 95% CI	upper 95% CI
TT	AA	44	55	1.00	-	-
TT	AG	33	55	1.31	0.73	2.37
TT	GG	14	19	1.10	0.49	2.45
CT	AA	0	9	1.00	-	-
CT	AG	24	38	0.00	0.00	-
CT	GG	8	15	0.00	0.00	-
CC	AA	0	0	1.00	-	-
CC	AG	0	0	-	-	-
CC	GG	2	4	-	-	-
Association of rs1805113 with rs11333758 (*p* = 0.012725)						
rs11333758	rs1805113	ctrl count	injured count	OR	lower 95% CI	upper 95% CI
AA	AA	26	41	1.00	-	-
AA	AG	35	53	0.85	0.44	1.66
AA	GG	21	19	0.50	0.22	1.12
A–	AA	14	21	0.79	0.33	1.85
A–	AG	20	34	1.01	0.47	2.13
A–	GG	3	17	3.46	0.91	13.16
– –	AA	4	2	0.26	0.04	1.56
– –	AG	2	6	1.92	0.35	10.47
– –	GG	0	2	-	0.00	-
Association of rs1805113 with injury after adjusting for rs11333758 genotype (*p* = 0.0020998)						
rs11333758	rs1805113	ctrl count	injured count	OR	lower 95% CI	upper 95% CI
AA	AA	26	41	1.00	-	-
AA	AG	35	53	0.85	0.44	1.66
AA	GG	21	19	0.50	0.22	1.12
A–	AA	14	21	1.00	-	-
A–	AG	20	34	1.28	0.53	3.11
A–	GG	3	17	4.41	1.07	18.25
– –	AA	4	2	1.00	-	-
– –	AG	2	6	7.34	0.69	77.92
– –	GG	0	2	-	0.00	-
Association of rs11333758 with injury after adjusting for rs1805113 genotype (*p* = 0.0039409)						
rs11333758	rs1805113	ctrl count	injured count	OR	lower 95% CI	upper 95% CI
AA	AA	26	41	1.00	-	-
A–	AA	14	21	0.79	0.33	1.85
– –	AA	4	2	0.26	0.04	1.56
AA	AG	35	53	1.00	-	-
A–	AG	20	34	1.18	0.58	2.39
– –	AG	2	6	2.25	0.42	12.08
AA	GG	21	19	1.00	-	-
A–	GG	3	17	6.94	1.73	27.95
– –	GG	0	2	-	0.00	-

The interaction between SNPs was examined using GLMs.

**Table 5:** Haplotype analysis for the two *TGFBR3* SNPs.

Haplotype		Association with Being in the Injured Group				
rs1805117	rs1805113	Frequency	OR	Lower 95% CI	Upper 95% CI	*p*-Value
T	A	0.552	1	reference haplotype		
T	G	0.283	1.13	0.79	1.61	0.495
C	A	0.0204	1.15	0.71	1.87	0.564
C	G	0.145	Inf	Inf	Inf	-

## Data Availability

The data presented in this study are available on request from the corresponding author. The data are not publicly available due to privacy/ethical restrictions.

## Supplementary Materials

The following supporting information can be downloaded at: https://www.mdpi.com/article/10.3390/jcm15010358/s1, Table S1: Non-reference allele frequencies for the study subgroups; Table S2: P-values for the Hardy-Weinberg equilibrium tests of the investigated polymorphisms; Table S3: Results of association analysis of selected SNPs with muscle injuries.

## Abbreviations

The following abbreviations are used in this manuscript:

3′UTR	3′ Untranslated Region
ACL	Anterior Cruciate Ligament
BMI	Body Mass Index
BMPs	Bone Morphogenetic Proteins
CI	Confidence Interval
CTGF	Connective Tissue Growth Factor
DNA	Deoxyribonucleic Acid
ECM	Muscle Extracellular Matrix
EM	Expectation-Maximization algorithm
GDF-8	Growth Differentiation Factor 8
GDFs	Growth and Differentiation Factors
GLM	Generalized Linear Model
GRCh38	Genome Reference Consortium Human build 38
HWE	Hardy–Weinberg Equilibrium
IPAQ	International Physical Activity Questionnaire
LTR	Log-Likelihood Ratio Test
MMPs	Matrix Metalloproteinases
MSTN	Myostatin
OR	Odds Ratio
PCR	Polymerase Chain Reaction
SNPs	Single Nucleotide Polymorphisms
STREGA	STrengthening the REporting of Genetic Association studies
TGF-β	Transforming Growth Factor-Beta
*TGFBI*	Transforming Growth Factor Beta Induced
*TGFBR3*	Transforming Growth Factor Beta Receptor 3

## References

[1] Gumucio J.P., Sugg K.B., Mendias C.L. (2015). TGF-β Superfamily Signaling in Muscle and Tendon Adaptation to Resistance Exercise. Exerc. Sport Sci. Rev..

[2] McPherron A.C., Lawler A.M., Lee S.J. (1997). Regulation of skeletal muscle mass in mice by a new TGF-β superfamily member. Nature.

[3] Morikawa M., Derynck R., Miyazono K. (2016). TGF- β and the TGF-β family: Context-dependent roles in cell and tissue physiology. Cold Spring Harb. Perspect. Biol..

[4] Deng Z., Fan T., Xiao C., Tian H., Zheng Y., Li C., He J. (2024). TGF-β signaling in health, disease, and therapeutics. Signal Transduct. Target. Ther..

[5] Soo C., Beanes S.R., Hu F.Y., Zhang X., Dang C., Chang G., Wang Y., Nishimura I., Freymiller E., Longaker M.T. (2003). Ontogenetic Transition in Fetal Wound Transforming Growth Factor-β Regulation Correlates with Collagen Organization. Am. J. Pathol..

[6] Gillies A.R., Chapman M.A., Bushong E.A., Deerinck T.J., Ellisman M.H., Lieber R.L. (2017). High resolution three-dimensional reconstruction of fibrotic skeletal muscle extracellular matrix. J. Physiol..

[7] Ismaeel A., Kim J.S., Kirk J.S., Smith R.S., Bohannon W.T., Koutakis P. (2019). Role of transforming growth factor-β in skeletal muscle fibrosis: A review. Int. J. Mol. Sci..

[8] Tekari A., Luginbuehl R., Hofstetter W., Egli R.J. (2015). Transforming growth factor beta signaling is essential for the autonomous formation of cartilage-like tissue by expanded chondrocytes. PLoS ONE.

[9] Ween M.P., Oehler M.K., Ricciardelli C. (2012). Transforming growth factor-beta-induced protein (TGFBI)/(βig-H3): A matrix protein with dual functions in ovarian cancer. Int. J. Mol. Sci..

[10] Laguette M.J.N., Barrow K., Firfirey F., Dlamini S., Saunders C.J., Dandara C., Gamieldien J., Collins M., September A.V. (2020). Exploring new genetic variants within COL5A1 intron 4-exon 5 region and TGF-β family with risk of anterior cruciate ligament ruptures. J. Orthop. Res..

[11] Posthumus M., Collins M., Cook J., Handley C.J., Ribbans W.J., Smith R.K.W., Schwellnus M.P., Raleigh S.M. (2010). Components of the transforming growth factor-β family and the pathogenesis of human achilles tendon pathology—A genetic association study. Rheumatology.

[12] Raleigh S.M., Posthumus M., O’Cuinneagain D., Van Der Merwe W., Collins M. (2013). The GDF5 gene and anterior cruciate ligament rupture. Int. J. Sports Med..

[13] Ginevičienė V., Jakaitienė A., Pranckevičienė E., Milašius K., Utkus A. (2021). Variants in the myostatin gene and physical performance phenotype of elite athletes. Genes.

[14] Leońska-Duniec A., Borczyk M., Korostyński M., Massidda M., Maculewicz E., Cięszczyk P. (2023). Genetic variants in myostatin and its receptors promote elite athlete status. BMC Genom..

[15] Jang J., Park S., Kim Y., Jung J., Lee J., Chang Y., Lee S.P., Park B.C., Wolfe R.R., Choi C.S. (2021). Myostatin Inhibition-Induced Increase in Muscle Mass and Strength Was Amplified by Resistance Exercise Training, and Dietary Essential Amino Acids Improved Muscle Quality in Mice. Nutrients.

[16] Biernacka A., Dobaczewski M., Frangogiannis N.G. (2011). TGF-β signaling in fibrosis. Growth Factors.

[17] Leask A., Abraham D.J. (2004). TGF-β signaling and the fibrotic response. FASEB J..

[18] Meng X.M., Nikolic-Paterson D.J., Lan H.Y. (2016). TGF-β: The master regulator of fibrosis. Nat. Rev. Nephrol..

[19] Kharraz Y., Guerra J., Pessina P., Serrano A.L., Muñoz-Cánoves P. (2014). Understanding the process of fibrosis in duchenne muscular dystrophy. BioMed Res. Int..

[20] Ceco E., McNally E.M. (2013). Modifying muscular dystrophy through transforming growth factor-β. FEBS J..

[21] Mendias C.L., Gumucio J.P., Davis M.E., Bromley C.W., Davis C.S., Brooks S.V. (2012). Transforming growth factor-beta induces skeletal muscle atrophy and fibrosis through the induction of atrogin-1 and scleraxis. Muscle Nerve.

[22] Dixon J.R., Selvaraj S., Yue F., Kim A., Li Y., Shen Y., Hu M., Liu J.S., Ren B. (2012). Topological domains in mammalian genomes identified by analysis of chromatin interactions. Nature.

[23] Kim J.H., Yu S.J., Park B.L., Cheong H.S., Pasaje C.F.A., Bae J.S., Lee H.S., Shin H.D., Kim Y.J. (2011). TGFBR3 polymorphisms and its haplotypes associated with chronic hepatitis B virus infection and age of hepatocellular carcinoma occurrence. Dig. Dis..

[24] Kim J.E., Jeong H.W., Nam J.O., Lee B.H., Choi J.Y., Park R.W., Park J.Y., Kim I.S. (2002). Identification of motifs in the fasciclin domains of the transforming growth factor-β-induced matrix protein βig-h3 that interact with the αvβ5 integrin. J. Biol. Chem..

