# Supplementary material for: The Transforming Growth Factor β Genes and Susceptibility to Musculoskeletal Injuries in a Physically Active Caucasian Cohort
Source: J Clin Med. 2026 Jan 3;15(1):358. doi: 10.3390/jcm15010358 (PMC12786868; doi:10.3390/jcm15010358)
Supplement: Supplementary file 1 [file jcm-15-00358-s001.zip › jcm-4015044-supplementary.pdf]

Supplementary Materials

Table S1. Non-reference allele frequencies for the study subgroups

rsID	GnomAD european (non-Finnish)	all participants	controls	injured group	one injury	more injuries	acl injury	muscle strain or tear	other injuries (sprains, twists or breaks)	female participants	male participants
rs1805117	0,17	0,16	0,14	0,18	0,16	0,17	0,21	0,17	0,20	0,15	0,17
rs1805113	0,44	0,42	0,42	0,43	0,43	0,42	0,48	0,41	0,40	0,40	0,44
rs11333758	0,19	0,20	0,20	0,24	0,21	0,23	0,23	0,24	0,27	0,20	0,23
rs1442	0,48	0,48	0,46	0,50	0,49	0,47	0,48	0,50	0,50	0,49	0,47

Table S2. *P*-values for the Hardy-Weinberg equilibrium tests of the investigated polymorphisms

rsID	all participants	controls	injured group	one injury	two injuries	acl injuries	muscle strain or tear	other injuries (sprains, twists or breaks)	female participants	male participants
rs1805117	0,32 (1)	1(1)	0,34 (1)	0,31 (1)	1 (1)	0,1 (0,4)	1 (1)	1 (1)	0,68 (1)	0,49 (1)
rs1805113	0,49 (1)	0,47 (1)	0,66 (1)	0,15 (0,6)	0,56 (1)	1 (1)	1 (1)	0,1 (0,4)	0,66 (1)	0,59 (1)
rs11333758	0,87 (1)	0,57 (1)	0,84 (1)	0,41 (1)	0,19 (0,76)	0,05 (0,20)	0,35 (1)	0,09 (0,36)	0,74 (1)	1 (1)

rs1442	0,5 (1)	0,72 (1)	0,2 (0,8)	1 (1)	0,26 (1)	0,43 (1)	0,12 (0,48)	**0,02 (0,08)**	0,29 (1)	0,15 (0,6)

Bonferroni-adjusted p-values are reported in brackets.

Table S3. Results of association analysis of selected SNPs with muscle injuries

**SNP**	**rs1805117**					
	**model**	**p-value**	**adjusted p-value**	**genotype**	**OR**	**95% confidence interval**
	codominant	0,51	1	TT	1	
				CT	1,34	0,81 - 2,23
				CC	1,30	0,23 - 7,36
	dominant	0,25	1	TT	1	
				CT - CC	1,34	0,81 - 2,20
	recessive	0,84	1	TT - CT	1	
				CC	1,2	0,21 - 6,72
	overdominant	0,26	1	TT - CC	1	
				CT	1,33	0,80 - 2,21
	additive	0,71	1	0,1,2	1,29	0,82 - 2,04
**SNP**	**rs1805113**					
	**model**	**p-value**	**adjusted p-value**	**genotype**	**OR**	**95% confidence interval**
	codominant	0,93	1	AA	1	
				AG	1,10	0,66 - 1,84
				GG	1,05	0,55 - 2,01
	dominant	0,73	1	AA	1	
				AG - GG	1,09	0,67 - 1,75
	recessive	0,98	1	AA - AG	1	
				GG	0,99	0,56 - 1,77
	overdominant	0,73	1	AA - GG	1	
				AG	1,08	0,69 - 1,71

	model	p-value	adjusted p-value	genotype	OR	95% confidence interval
	additive	0,83	1	0,1,2	1,04	0,75 - 1,42
**SNP**	**rs11333758**					
	**model**	**p-value**	**adjusted p-value**	**genotype**	**OR**	**95% confidence interval**
	codominant	0,40	1	AA	1	
				A–	1,40	0,86 - 2,29
				––	1,18	0,41 - 3,42
	dominant	0,19	0,74	AA	1	
				A– - ––	1,37	0,86 - 2,19
	recessive	0,92	1	AA - A–	1	
				––	1,05	0,37 - 3
	overdominant	0,19	0,74	AA - ––	1	
				A–	1,38	0,85 - 2,25
	additive	0,26	1	0,1,2	1,25	0,85 - 1,86
**SNP**	**rs1442**					
	**model**	**p-value**	**adjusted p-value**	**genotype**	**OR**	**95% confidence interval**
	codominant	0,36	1	CC	1	
				CG	0,96	0,56 - 1,64
				GG	1,43	0,75 - 2,73
	dominant	0,73	1	CC	1	
				CG - GG	1,09	0,66 - 1,82
	recessive	0,16	0,63	CC - CG	1	
				GG	1,48	0,86 - 2,54
	overdominant	0,36	1	CC - GG	1	
				CG	0,81	0,51 - 1,28
	additive	0,29	1	0,1,2	1,18	0,86 - 1,63

OR - Odds Ratio
